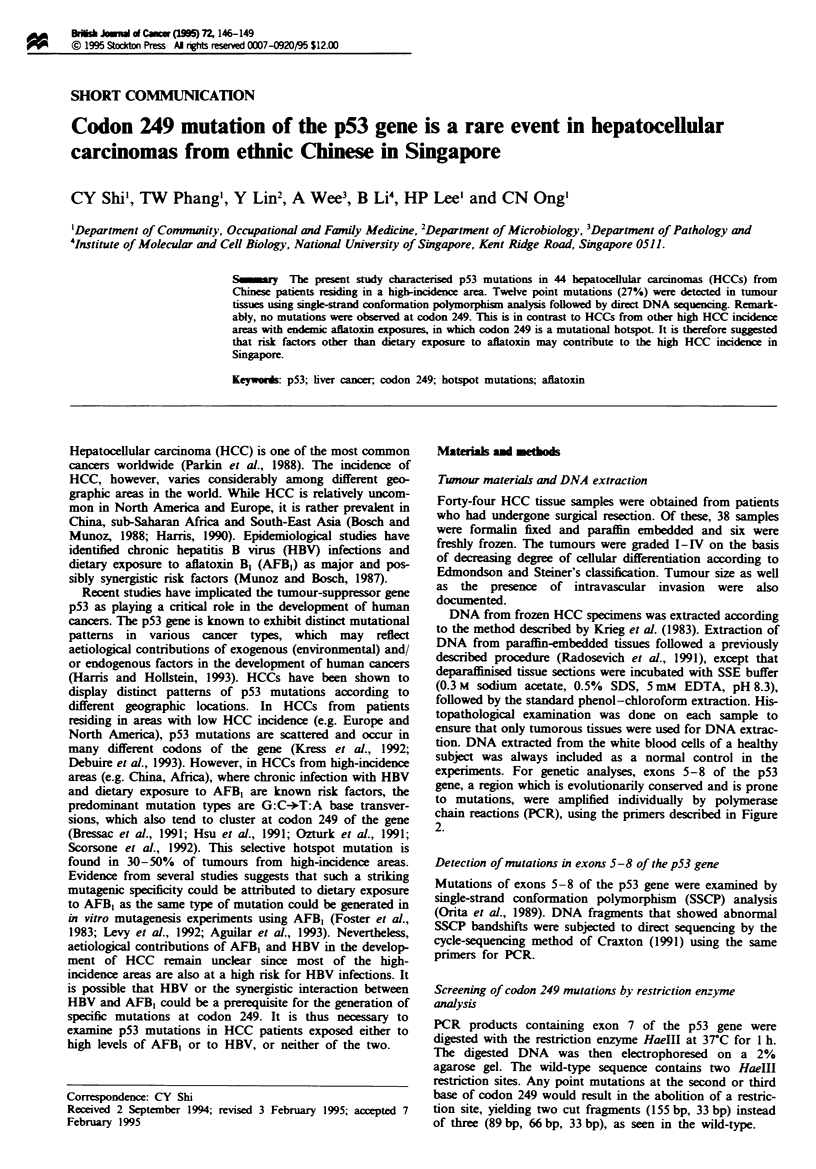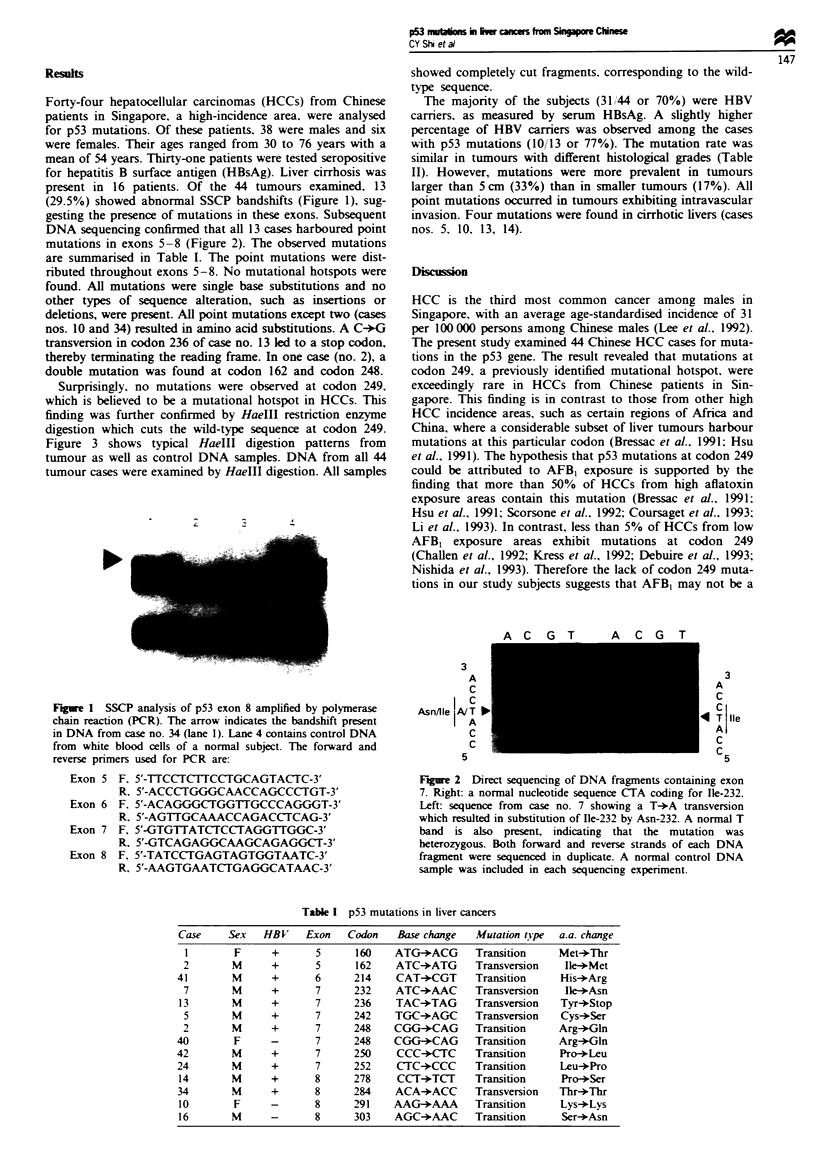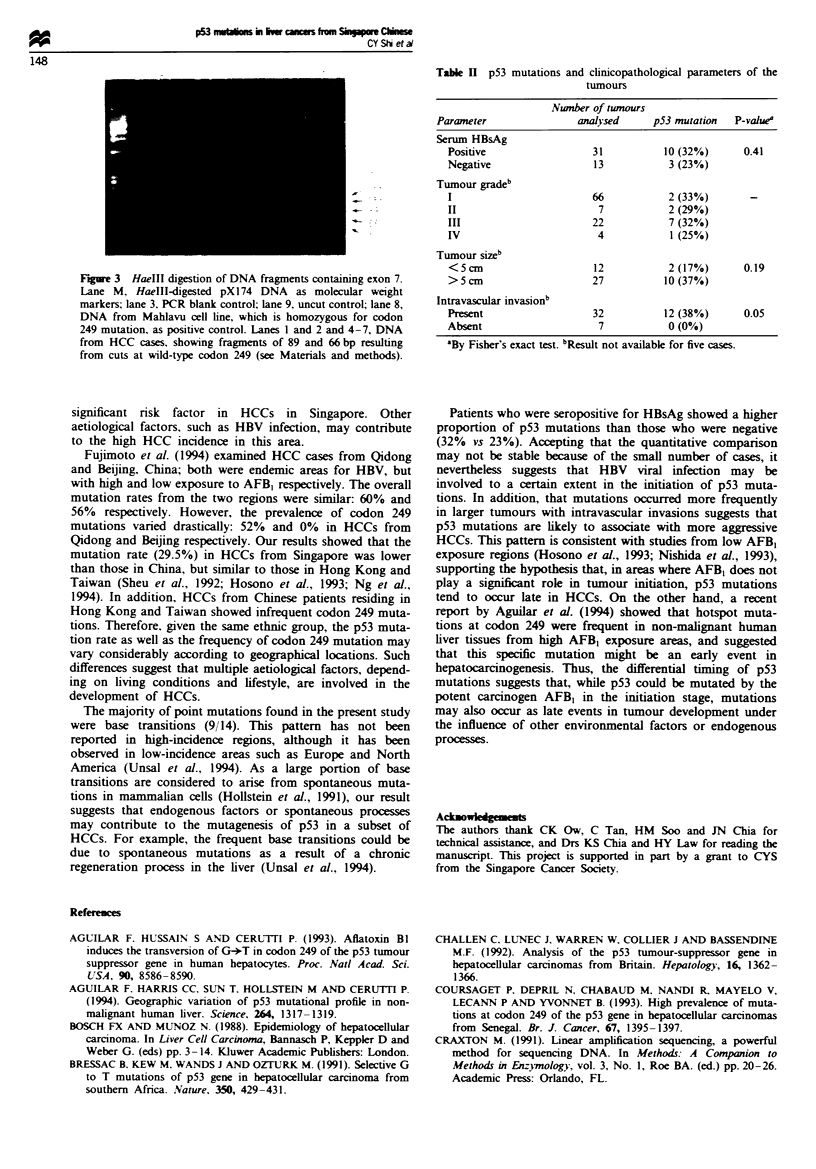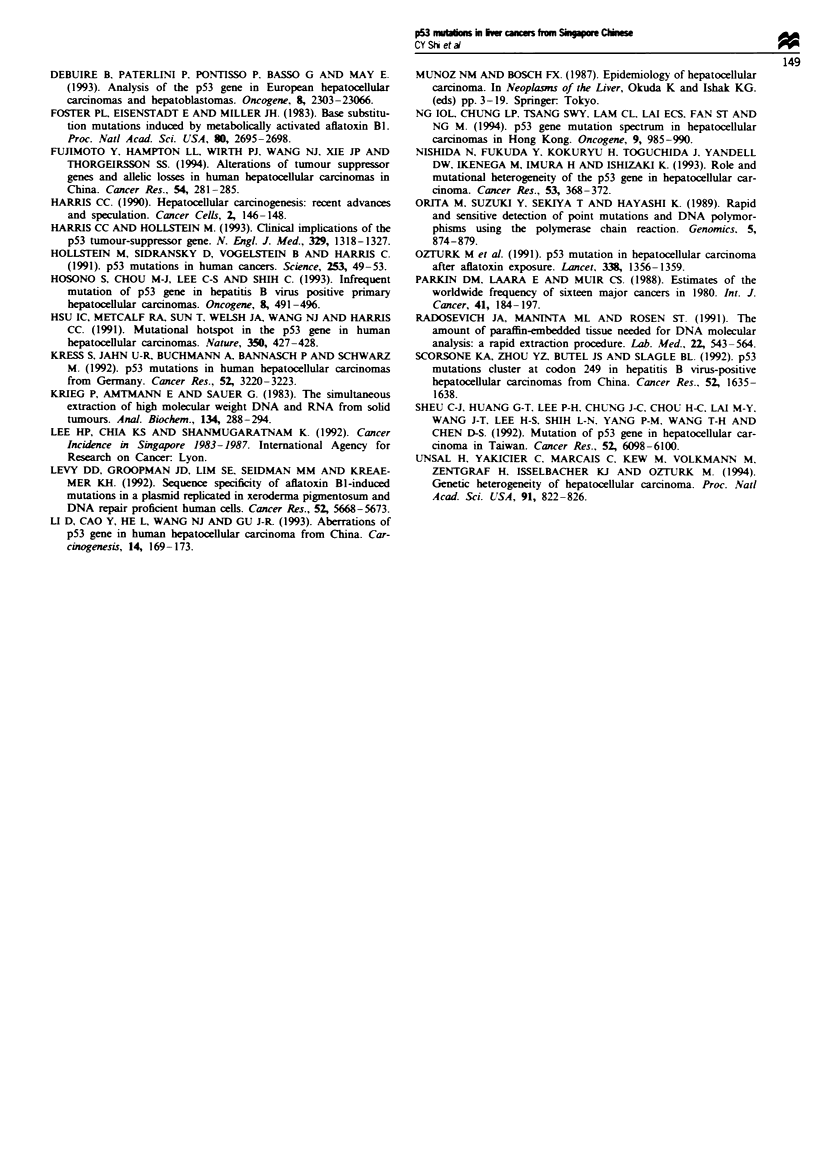# Codon 249 mutation of the p53 gene is a rare event in hepatocellular carcinomas from ethnic Chinese in Singapore.

**DOI:** 10.1038/bjc.1995.291

**Published:** 1995-07

**Authors:** C. Y. Shi, T. W. Phang, Y. Lin, A. Wee, B. Li, H. P. Lee, C. N. Ong

**Affiliations:** Department of Community, Occupational and Family Medicine, National University of Singapore.

## Abstract

**Images:**


© 1995 Stockton Press All rights reserved 0007-0920/95 $12.00

SHORT COMMUNICATION

### Codon 249 mutation of the p53 gene is a rare event in hepatocellular carcinomas from ethnic Chinese in Singapore

CY Shi[1], TW Phang[1], Y Lin[2], A Wee[3], B Li[4], HP Lee[1] and CN Ong[1]

[1]*Department of Community, Occupational and Family Medicine,* [2]*Department of Microbiology,* [3]*Department of Pathology and* [4]*Institute of Molecular and Cell Biology, National University of Singapore, Kent Ridge Road, Singapore 0511.*

**Summary** The present study characterised p53 mutations in 44 hepatocellular carcinomas (HCCs) from Chinese patients residing in a high-incidence area. Twelve point mutations (27%) were detected in tumour tissues using single-strand conformation polymorphism analysis followed by direct DNA sequencing. Remarkably, no mutations were observed at codon 249. This is in contrast to HCCs from other high HCC incidence areas with endemic aflatoxin exposures, in which codon 249 is a mutational hotspot. It is therefore suggested that risk factors other than dietary exposure to aflatoxin may contribute to the high HCC incidence in Singapore.

**Keywords**: p53; liver cancer; codon 249; hotspot mutations; aflatoxin

Hepatocellular carcinoma (HCC) is one of the most common cancers worldwide (Parkin *et al.*, 1988). The incidence of HCC, however, varies considerably among different geographic areas in the world. While HCC is relatively uncommon in North America and Europe, it is rather prevalent in China, sub-Saharan Africa and South-East Asia (Bosch and Munoz, 1988; Harris, 1990). Epidemiological studies have identified chronic hepatitis B virus (HBV) infections and dietary exposure to aflatoxin $B_1$ ($AFB_1$) as major and possibly synergistic risk factors (Munoz and Bosch, 1987).

Recent studies have implicated the tumour-suppressor gene p53 as playing a critical role in the development of human cancers. The p53 gene is known to exhibit distinct mutational patterns in various cancer types, which may reflect aetiological contributions of exogenous (environmental) and/or endogenous factors in the development of human cancers (Harris and Hollstein, 1993). HCCs have been shown to display distinct patterns of p53 mutations according to different geographic locations. In HCCs from patients residing in areas with low HCC incidence (e.g. Europe and North America), p53 mutations are scattered and occur in many different codons of the gene (Kress *et al.*, 1992; Debuire *et al.*, 1993). However, in HCCs from high-incidence areas (e.g. China, Africa), where chronic infection with HBV and dietary exposure to $AFB_1$ are known risk factors, the predominant mutation types are G:C→T:A base transversions, which also tend to cluster at codon 249 of the gene (Bressac *et al.*, 1991; Hsu *et al.*, 1991; Ozturk *et al.*, 1991; Scorsone *et al.*, 1992). This selective hotspot mutation is found in 30-50% of tumours from high-incidence areas. Evidence from several studies suggests that such a striking mutagenic specificity could be attributed to dietary exposure to $AFB_1$ as the same type of mutation could be generated in *in vitro* mutagenesis experiments using $AFB_1$ (Foster *et al.*, 1983; Levy *et al.*, 1992; Aguilar *et al.*, 1993). Nevertheless, aetiological contributions of $AFB_1$ and HBV in the development of HCC remain unclear since most of the high-incidence areas are also at a high risk for HBV infections. It is possible that HBV or the synergistic interaction between HBV and $AFB_1$ could be a prerequisite for the generation of specific mutations at codon 249. It is thus necessary to examine p53 mutations in HCC patients exposed either to high levels of $AFB_1$ or to HBV, or neither of the two.

Correspondence: CY Shi
Received 2 September 1994; revised 3 February 1995; accepted 7 February 1995

### Materials and methods

### *Tumour materials and DNA extraction*

Forty-four HCC tissue samples were obtained from patients who had undergone surgical resection. Of these, 38 samples were formalin fixed and paraffin embedded and six were freshly frozen. The tumours were graded I-IV on the basis of decreasing degree of cellular differentiation according to Edmondson and Steiner's classification. Tumour size as well as the presence of intravascular invasion were also documented.

DNA from frozen HCC specimens was extracted according to the method described by Krieg *et al.* (1983). Extraction of DNA from paraffin-embedded tissues followed a previously described procedure (Radosevich *et al.*, 1991), except that deparaffinised tissue sections were incubated with SSE buffer (0.3 M sodium acetate, 0.5% SDS, 5 mM EDTA, pH 8.3), followed by the standard phenol-chloroform extraction. Histopathological examination was done on each sample to ensure that only tumorous tissues were used for DNA extraction. DNA extracted from the white blood cells of a healthy subject was always included as a normal control in the experiments. For genetic analyses, exons 5-8 of the p53 gene, a region which is evolutionarily conserved and is prone to mutations, were amplified individually by polymerase chain reactions (PCR), using the primers described in Figure 2.

### *Detection of mutations in exons 5-8 of the p53 gene*

Mutations of exons 5-8 of the p53 gene were examined by single-strand conformation polymorphism (SSCP) analysis (Orita *et al.*, 1989). DNA fragments that showed abnormal SSCP bandshifts were subjected to direct sequencing by the cycle-sequencing method of Craxton (1991) using the same primers for PCR.

### *Screening of codon 249 mutations by restriction enzyme analysis*

PCR products containing exon 7 of the p53 gene were digested with the restriction enzyme *Hae*III at 37°C for 1 h. The digested DNA was then electrophoresed on a 2% agarose gel. The wild-type sequence contains two *Hae*III restriction sites. Any point mutations at the second or third base of codon 249 would result in the abolition of a restriction site, yielding two cut fragments (155 bp, 33 bp) instead of three (89 bp, 66 bp, 33 bp), as seen in the wild-type.

### Results

Forty-four hepatocellular carcinomas (HCCs) from Chinese patients in Singapore, a high-incidence area, were analysed for p53 mutations. Of these patients, 38 were males and six were females. Their ages ranged from 30 to 76 years with a mean of 54 years. Thirty-one patients were tested seropositive for hepatitis B surface antigen (HBsAg). Liver cirrhosis was present in 16 patients. Of the 44 tumours examined, 13 (29.5%) showed abnormal SSCP bandshifts (Figure 1), suggesting the presence of mutations in these exons. Subsequent DNA sequencing confirmed that all 13 cases harboured point mutations in exons 5–8 (Figure 2). The observed mutations are summarised in Table I. The point mutations were distributed throughout exons 5–8. No mutational hotspots were found. All mutations were single base substitutions and no other types of sequence alteration, such as insertions or deletions, were present. All point mutations except two (cases nos. 10 and 34) resulted in amino acid substitutions. A C→G transversion in codon 236 of case no. 13 led to a stop codon, thereby terminating the reading frame. In one case (no. 2), a double mutation was found at codon 162 and codon 248.

Surprisingly, no mutations were observed at codon 249, which is believed to be a mutational hotspot in HCCs. This finding was further confirmed by *Hae*III restriction enzyme digestion which cuts the wild-type sequence at codon 249. Figure 3 shows typical *Hae*III digestion patterns from tumour as well as control DNA samples. DNA from all 44 tumour cases were examined by *Hae*III digestion. All samples showed completely cut fragments, corresponding to the wild-type sequence.

The majority of the subjects (31/44 or 70%) were HBV carriers, as measured by serum HBsAg. A slightly higher percentage of HBV carriers was observed among the cases with p53 mutations (10/13 or 77%). The mutation rate was similar in tumours with different histological grades (Table II). However, mutations were more prevalent in tumours larger than 5 cm (33%) than in smaller tumours (17%). All point mutations occurred in tumours exhibiting intravascular invasion. Four mutations were found in cirrhotic livers (cases nos. 5, 10, 13, 14).

**Figure 1** SSCP analysis of p53 exon 8 amplified by polymerase chain reaction (PCR). The arrow indicates the bandshift present in DNA from case no. 34 (lane 1). Lane 4 contains control DNA from white blood cells of a normal subject. The forward and reverse primers used for PCR are:

| | | |
|---|---|---|
| Exon 5 | F, | 5′-TTCCTCTTCCTGCAGTACTC-3′ |
| | R, | 5′-ACCCTGGGCAACCAGCCCTGT-3′ |
| Exon 6 | F, | 5′-ACAGGGCTGGTTGCCCAGGGT-3′ |
| | R, | 5′-AGTTGCAAACCAGACCTCAG-3′ |
| Exon 7 | F, | 5′-GTGTTATCTCCTAGGTTGGC-3′ |
| | R, | 5′-GTCAGAGGCAAGCAGAGGCT-3′ |
| Exon 8 | F, | 5′-TATCCTGAGTAGTGGTAATC-3′ |
| | R, | 5′-AAGTGAATCTGAGGCATAAC-3′ |

### Discussion

HCC is the third most common cancer among males in Singapore, with an average age-standardised incidence of 31 per 100 000 persons among Chinese males (Lee *et al.*, 1992). The present study examined 44 Chinese HCC cases for mutations in the p53 gene. The result revealed that mutations at codon 249, a previously identified mutational hotspot, were exceedingly rare in HCCs from Chinese patients in Singapore. This finding is in contrast to those from other high HCC incidence areas, such as certain regions of Africa and China, where a considerable subset of liver tumours harbour mutations at this particular codon (Bressac *et al.*, 1991; Hsu *et al.*, 1991). The hypothesis that p53 mutations at codon 249 could be attributed to $AFB_1$ exposure is supported by the finding that more than 50% of HCCs from high aflatoxin exposure areas contain this mutation (Bressac *et al.*, 1991; Hsu *et al.*, 1991; Scorsone *et al.*, 1992; Coursaget *et al.*, 1993; Li *et al.*, 1993). In contrast, less than 5% of HCCs from low $AFB_1$ exposure areas exhibit mutations at codon 249 (Challen *et al.*, 1992; Kress *et al.*, 1992; Debuire *et al.*, 1993; Nishida *et al.*, 1993). Therefore the lack of codon 249 mutations in our study subjects suggests that $AFB_1$ may not be a

**Figure 2** Direct sequencing of DNA fragments containing exon 7. Right: a normal nucleotide sequence CTA coding for Ile-232. Left: sequence from case no. 7 showing a T→A transversion which resulted in substitution of Ile-232 by Asn-232. A normal T band is also present, indicating that the mutation was heterozygous. Both forward and reverse strands of each DNA fragment were sequenced in duplicate. A normal control DNA sample was included in each sequencing experiment.

**Table I** p53 mutations in liver cancers

| *Case* | *Sex* | *HBV* | *Exon* | *Codon* | *Base change* | *Mutation type* | *a.a. change* |
|---|---|---|---|---|---|---|---|
| 1 | F | + | 5 | 160 | ATG→ACG | Transition | Met→Thr |
| 2 | M | + | 5 | 162 | ATC→ATG | Transversion | Ile→Met |
| 41 | M | + | 6 | 214 | CAT→CGT | Transition | His→Arg |
| 7 | M | + | 7 | 232 | ATC→AAC | Transversion | Ile→Asn |
| 13 | M | + | 7 | 236 | TAC→TAG | Transversion | Tyr→Stop |
| 5 | M | + | 7 | 242 | TGC→AGC | Transversion | Cys→Ser |
| 2 | M | + | 7 | 248 | CGG→CAG | Transition | Arg→Gln |
| 40 | F | – | 7 | 248 | CGG→CAG | Transition | Arg→Gln |
| 42 | M | + | 7 | 250 | CCC→CTC | Transition | Pro→Leu |
| 24 | M | + | 7 | 252 | CTC→CCC | Transition | Leu→Pro |
| 14 | M | + | 8 | 278 | CCT→TCT | Transition | Pro→Ser |
| 34 | M | + | 8 | 284 | ACA→ACC | Transversion | Thr→Thr |
| 10 | F | – | 8 | 291 | AAG→AAA | Transition | Lys→Lys |
| 16 | M | – | 8 | 303 | AGC→AAC | Transition | Ser→Asn |

**Figure 3** *Hae*III digestion of DNA fragments containing exon 7. Lane M, *Hae*III-digested pX174 DNA as molecular weight markers; lane 3, PCR blank control; lane 9, uncut control; lane 8, DNA from Mahlavu cell line, which is homozygous for codon 249 mutation, as positive control. Lanes 1 and 2 and 4–7, DNA from HCC cases, showing fragments of 89 and 66 bp resulting from cuts at wild-type codon 249 (see Materials and methods).

**Table II** p53 mutations and clinicopathological parameters of the tumours

| *Parameter* | *Number of tumours analysed* | *p53 mutation* | P-*value*[a] |
|---|---|---|---|
| Serum HBsAg | | | |
| Positive | 31 | 10 (32%) | 0.41 |
| Negative | 13 | 3 (23%) | |
| Tumour grade[b] | | | |
| I | 66 | 2 (33%) | – |
| II | 7 | 2 (29%) | |
| III | 22 | 7 (32%) | |
| IV | 4 | 1 (25%) | |
| Tumour size[b] | | | |
| <5 cm | 12 | 2 (17%) | 0.19 |
| >5 cm | 27 | 10 (37%) | |
| Intravascular invasion[b] | | | |
| Present | 32 | 12 (38%) | 0.05 |
| Absent | 7 | 0 (0%) | |

[a]By Fisher's exact test. [b]Result not available for five cases.

significant risk factor in HCCs in Singapore. Other aetiological factors, such as HBV infection, may contribute to the high HCC incidence in this area.

Fujimoto *et al.* (1994) examined HCC cases from Qidong and Beijing, China; both were endemic areas for HBV, but with high and low exposure to $AFB_1$ respectively. The overall mutation rates from the two regions were similar: 60% and 56% respectively. However, the prevalence of codon 249 mutations varied drastically: 52% and 0% in HCCs from Qidong and Beijing respectively. Our results showed that the mutation rate (29.5%) in HCCs from Singapore was lower than those in China, but similar to those in Hong Kong and Taiwan (Sheu *et al.*, 1992; Hosono *et al.*, 1993; Ng *et al.*, 1994). In addition, HCCs from Chinese patients residing in Hong Kong and Taiwan showed infrequent codon 249 mutations. Therefore, given the same ethnic group, the p53 mutation rate as well as the frequency of codon 249 mutation may vary considerably according to geographical locations. Such differences suggest that multiple aetiological factors, depending on living conditions and lifestyle, are involved in the development of HCCs.

The majority of point mutations found in the present study were base transitions (9/14). This pattern has not been reported in high-incidence regions, although it has been observed in low-incidence areas such as Europe and North America (Unsal *et al.*, 1994). As a large portion of base transitions are considered to arise from spontaneous mutations in mammalian cells (Hollstein *et al.*, 1991), our result suggests that endogenous factors or spontaneous processes may contribute to the mutagenesis of p53 in a subset of HCCs. For example, the frequent base transitions could be due to spontaneous mutations as a result of a chronic regeneration process in the liver (Unsal *et al.*, 1994).

Patients who were seropositive for HBsAg showed a higher proportion of p53 mutations than those who were negative (32% *vs* 23%). Accepting that the quantitative comparison may not be stable because of the small number of cases, it nevertheless suggests that HBV viral infection may be involved to a certain extent in the initiation of p53 mutations. In addition, that mutations occurred more frequently in larger tumours with intravascular invasions suggests that p53 mutations are likely to associate with more aggressive HCCs. This pattern is consistent with studies from low $AFB_1$ exposure regions (Hosono *et al.*, 1993; Nishida *et al.*, 1993), supporting the hypothesis that, in areas where $AFB_1$ does not play a significant role in tumour initiation, p53 mutations tend to occur late in HCCs. On the other hand, a recent report by Aguilar *et al.* (1994) showed that hotspot mutations at codon 249 were frequent in non-malignant human liver tissues from high $AFB_1$ exposure areas, and suggested that this specific mutation might be an early event in hepatocarcinogenesis. Thus, the differential timing of p53 mutations suggests that, while p53 could be mutated by the potent carcinogen $AFB_1$ in the initiation stage, mutations may also occur as late events in tumour development under the influence of other environmental factors or endogenous processes.

**Acknowledgements**
The authors thank CK Ow, C Tan, HM Soo and JN Chia for technical assistance, and Drs KS Chia and HY Law for reading the manuscript. This project is supported in part by a grant to CYS from the Singapore Cancer Society.

**References**

AGUILAR F, HUSSAIN S AND CERUTTI P. (1993). Aflatoxin B1 induces the transversion of G→T in codon 249 of the p53 tumour suppressor gene in human hepatocytes. *Proc. Natl Acad. Sci. USA*, **90**, 8586–8590.
AGUILAR F, HARRIS CC, SUN T, HOLLSTEIN M AND CERUTTI P. (1994). Geographic variation of p53 mutational profile in non-malignant human liver. *Science*, **264**, 1317–1319.
BOSCH FX AND MUNOZ N. (1988). Epidemiology of hepatocellular carcinoma. In *Liver Cell Carcinoma*, Bannasch P, Keppler D and Weber G. (eds) pp. 3–14. Kluwer Academic Publishers: London.
BRESSAC B, KEW M, WANDS J AND OZTURK M. (1991). Selective G to T mutations of p53 gene in hepatocellular carcinoma from southern Africa. *Nature*, **350**, 429–431.
CHALLEN C, LUNEC J, WARREN W, COLLIER J AND BASSENDINE M.F. (1992). Analysis of the p53 tumour-suppressor gene in hepatocellular carcinomas from Britain. *Hepatology*, **16**, 1362–1366.
COURSAGET P, DEPRIL N, CHABAUD M, NANDI R, MAYELO V, LECANN P AND YVONNET B. (1993). High prevalence of mutations at codon 249 of the p53 gene in hepatocellular carcinomas from Senegal. *Br. J. Cancer*, **67**, 1395–1397.
CRAXTON M. (1991). Linear amplification sequencing, a powerful method for sequencing DNA. In *Methods: A Companion to Methods in Enzymology*, vol. 3, No. 1, Roe BA. (ed.) pp. 20–26. Academic Press: Orlando, FL.

DEBUIRE B, PATERLINI P, PONTISSO P, BASSO G AND MAY E. (1993). Analysis of the p53 gene in European hepatocellular carcinomas and hepatoblastomas. *Oncogene*, **8**, 2303–23066.

FOSTER PL, EISENSTADT E AND MILLER JH. (1983). Base substitution mutations induced by metabolically activated aflatoxin B1. *Proc. Natl Acad. Sci. USA*, **80**, 2695–2698.

FUJIMOTO Y, HAMPTON LL, WIRTH PJ, WANG NJ, XIE JP AND THORGEIRSSON SS. (1994). Alterations of tumour suppressor genes and allelic losses in human hepatocellular carcinomas in China. *Cancer Res.*, **54**, 281–285.

HARRIS CC. (1990). Hepatocellular carcinogenesis: recent advances and speculation. *Cancer Cells*, **2**, 146–148.

HARRIS CC AND HOLLSTEIN M. (1993). Clinical implications of the p53 tumour-suppressor gene. *N. Engl. J. Med.*, **329**, 1318–1327.

HOLLSTEIN M, SIDRANSKY D, VOGELSTEIN B AND HARRIS C. (1991). p53 mutations in human cancers. *Science*, **253**, 49–53.

HOSONO S, CHOU M-J, LEE C-S AND SHIH C. (1993). Infrequent mutation of p53 gene in hepatitis B virus positive primary hepatocellular carcinomas. *Oncogene*, **8**, 491–496.

HSU IC, METCALF RA, SUN T, WELSH JA, WANG NJ AND HARRIS CC. (1991). Mutational hotspot in the p53 gene in human hepatocellular carcinomas. *Nature*, **350**, 427–428.

KRESS S, JAHN U-R, BUCHMANN A, BANNASCH P AND SCHWARZ M. (1992). p53 mutations in human hepatocellular carcinomas from Germany. *Cancer Res.*, **52**, 3220–3223.

KRIEG P, AMTMANN E AND SAUER G. (1983). The simultaneous extraction of high molecular weight DNA and RNA from solid tumours. *Anal. Biochem.*, **134**, 288–294.

LEE HP, CHIA KS AND SHANMUGARATNAM K. (1992). *Cancer Incidence in Singapore 1983–1987*. International Agency for Research on Cancer: Lyon.

LEVY DD, GROOPMAN JD, LIM SE, SEIDMAN MM AND KREAEMER KH. (1992). Sequence specificity of aflatoxin B1-induced mutations in a plasmid replicated in xeroderma pigmentosum and DNA repair proficient human cells. *Cancer Res.*, **52**, 5668–5673.

LI D, CAO Y, HE L, WANG NJ AND GU J-R. (1993). Aberrations of p53 gene in human hepatocellular carcinoma from China. *Carcinogenesis*, **14**, 169–173.

MUNOZ NM AND BOSCH FX. (1987). Epidemiology of hepatocellular carcinoma. In *Neoplasms of the Liver*, Okuda K and Ishak KG. (eds) pp. 3–19. Springer: Tokyo.

NG IOL, CHUNG LP, TSANG SWY, LAM CL, LAI ECS, FAN ST AND NG M. (1994). p53 gene mutation spectrum in hepatocellular carcinomas in Hong Kong. *Oncogene*, **9**, 985–990.

NISHIDA N, FUKUDA Y, KOKURYU H, TOGUCHIDA J, YANDELL DW, IKENEGA M, IMURA H AND ISHIZAKI K. (1993). Role and mutational heterogeneity of the p53 gene in hepatocellular carcinoma. *Cancer Res.*, **53**, 368–372.

ORITA M, SUZUKI Y, SEKIYA T AND HAYASHI K. (1989). Rapid and sensitive detection of point mutations and DNA polymorphisms using the polymerase chain reaction. *Genomics*, **5**, 874–879.

OZTURK M *et al.* (1991). p53 mutation in hepatocellular carcinoma after aflatoxin exposure. *Lancet*, **338**, 1356–1359.

PARKIN DM, LAARA E AND MUIR CS. (1988). Estimates of the worldwide frequency of sixteen major cancers in 1980. *Int. J. Cancer*, **41**, 184–197.

RADOSEVICH JA, MANINTA ML AND ROSEN ST. (1991). The amount of paraffin-embedded tissue needed for DNA molecular analysis: a rapid extraction procedure. *Lab. Med.*, **22**, 543–564.

SCORSONE KA, ZHOU YZ, BUTEL JS AND SLAGLE BL. (1992). p53 mutations cluster at codon 249 in hepatitis B virus-positive hepatocellular carcinomas from China. *Cancer Res.*, **52**, 1635–1638.

SHEU C-J, HUANG G-T, LEE P-H, CHUNG J-C, CHOU H-C, LAI M-Y, WANG J-T, LEE H-S, SHIH L-N, YANG P-M, WANG T-H AND CHEN D-S. (1992). Mutation of p53 gene in hepatocellular carcinoma in Taiwan. *Cancer Res.*, **52**, 6098–6100.

UNSAL H, YAKICIER C, MARCAIS C, KEW M, VOLKMANN M, ZENTGRAF H, ISSELBACHER KJ AND OZTURK M. (1994). Genetic heterogeneity of hepatocellular carcinoma. *Proc. Natl Acad. Sci. USA*, **91**, 822–826.